# The Role of microRNA Let-7d in Female Malignancies and Diseases of the Female Reproductive Tract

**DOI:** 10.3390/ijms22147359

**Published:** 2021-07-08

**Authors:** Chiara De Santis, Martin Götte

**Affiliations:** Department of Gynecology and Obstetrics, Münster University Hospital, Albert-Schweitzer—Campus 1, D11, 48149 Münster, Germany; Chiara.DeSantis@ukmuenster.de

**Keywords:** microRNAs, let-7, breast cancer, ovarian cancer, endometriosis, preeclampsia, fetal growth restriction, therapeutic resistance, biomarker

## Abstract

microRNAs are small noncoding RNAs that regulate gene expression at the posttranscriptional level. Let-7d is a microRNA of the conserved let-7 family that is dysregulated in female malignancies including breast cancer, ovarian cancer, endometrial cancer, and cervical cancer. Moreover, a dysregulation is observed in endometriosis and pregnancy-associated diseases such as preeclampsia and fetal growth restriction. Let-7d expression is regulated by cytokines and steroids, involving transcriptional regulation by OCT4, MYC and p53, as well as posttranscriptional regulation via LIN28 and ADAR. By downregulating a wide range of relevant mRNA targets, let-7d affects cellular processes that drive disease progression such as cell proliferation, apoptosis (resistance), angiogenesis and immune cell function. In an oncological context, let-7d has a tumor-suppressive function, although some of its functions are context-dependent. Notably, its expression is associated with improved therapeutic responses to chemotherapy in breast and ovarian cancer. Studies in mouse models have furthermore revealed important roles in uterine development and function, with implications for obstetric diseases. Apart from a possible utility as a diagnostic blood-based biomarker, pharmacological modulation of let-7d emerges as a promising therapeutic concept in a variety of female disease conditions.

## 1. A Primer to MicroRNAs

MicroRNAs are a group of endogenous small single-stranded non-coding RNA molecules of 19–24 nucleotides that regulate gene expression at the posttranscriptional level [1,2,3,4]. Most microRNA encoding genes are inserted in intronic regions of protein-encoding genes. They are either transcribed under the control of their own promoters or they share a promoter with mRNA. In the genome, microRNAs can be organised as microRNA clusters [1]. After transcription by RNA polymerase II in the nucleus, primary micro-RNA (pri-microRNA), comprising about 500–3000 nucleotides, is processed into stem-loop precursor microRNA (pre-microRNA) by RNA III endonuclease Drosha and the dsRNA binding protein DGCR8/Pasha (a complex known as microprocessor) [4]. After translocation into the cytoplasm via the transport system exportin-5 in a Ran-GTP-dependent process, pre-microRNA is converted into a ~20-bp microRNA duplex by RNAse III-enzyme Dicer characterized by the presence of a 5′ phosphate and a two nucleotide 3′ overhang on each end [4,5]. Of the microRNA double strand, the mature microRNA enters the guide-strand channel of an Argonaute protein thus forming a silencing complex, whereas the complementary microRNA* strand is degraded. While binding to 3′-untranslated regions (UTR) of complement target mRNAs as part of the RNA-induced silencing complex (RISC) microRNAs promote either their degradation or translational repression, depending on the degree of complementarity between the so-called seed-sequence of the mature microRNA and the corresponding complementary target region in the cognate mRNA [2,3,6]. MicroRNAs are expressed in tissue and cell-type-specific expression profiles and they are implicated in physiologic and pathologic processes, including the cell cycle, apoptosis, proliferation, differentiation, metabolic pathways and cell response to various types of stress [7,8,9,10].

## 2. Let-7d—A Member of the Conserved Let-7 Family of microRNAs

The human lethal-7 (let-7) family has an important role in the regulation of cell proliferation and carcinogenesis. It includes 13 members in 9 loci on 7 chromosomes, and represents an evolutionarily conserved microRNA family [11].

**Figure 1:**
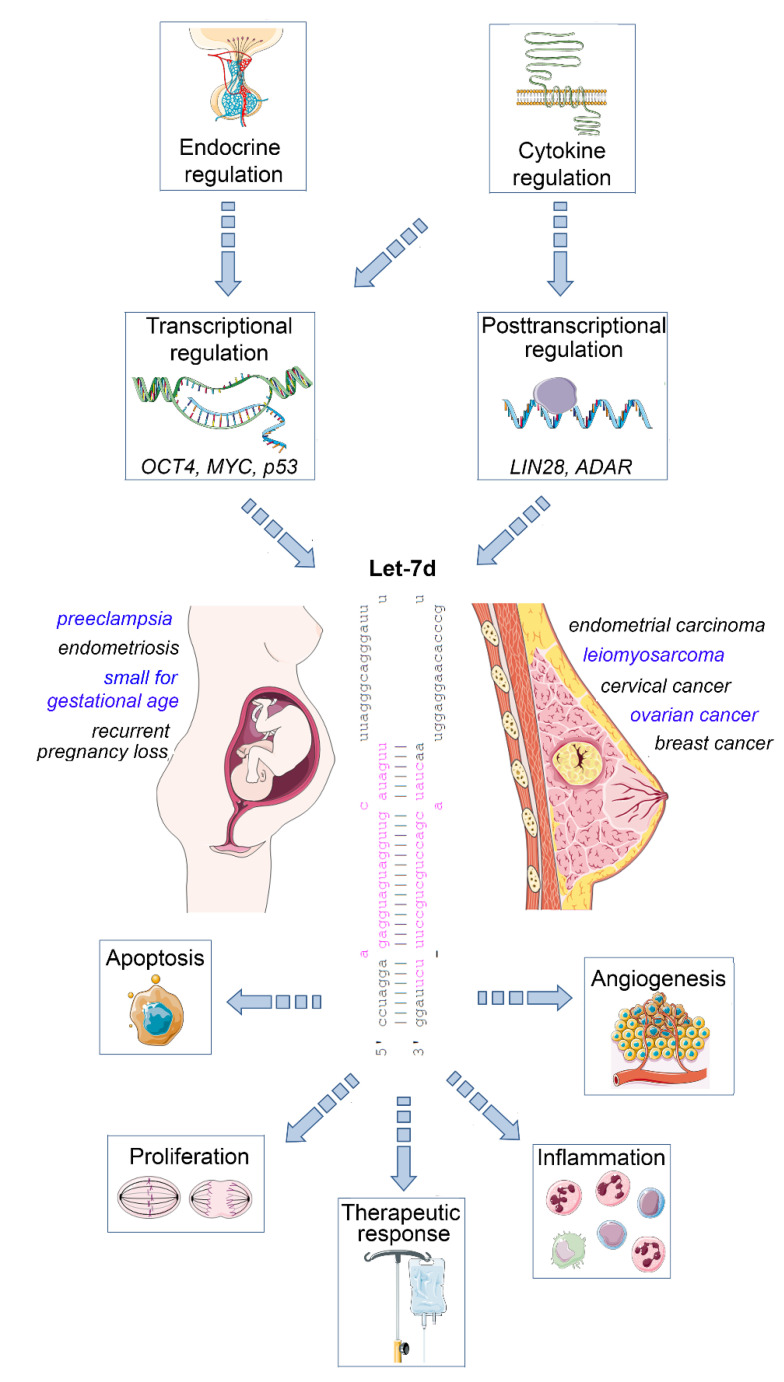
Impact of let-7d on female malignancies and diseases of the female reproductive tract. Let-7d expression is dysregulated in a variety of gynaecological and obstetric disorders. Its expression is regulated in a hormone- and cytokine-dependent manner involving both transcriptional and post-transcriptional mechanisms. Let-7d targets key mRNAs involved in the regulation of cell proliferation, apoptosis, angiogenesis and immune cell function, thereby modulating disease progression and therapeutic response. See text for details. The Let-7d sequence was retrieved from miRbase [24]. This figure was designed using elements of the free web resource Smart Servier Medical Art (https://smart.servier.com/ (accessed on 1 June 2021)).

The mature let-7 family members are the most common among all microRNAs [4]. Let-7d is one of the members of this family, and is located within the let-7a-1/let-7f-1/let-7d cluster on chromosome 9, which is transcribed as a single polycistronic transcript [11]. Let-7 expression is controlled by different transcriptional and post-transcriptional processes, including transcriptional regulation by OCT4, MYC and mutant p53 [11,12], and posttranscriptional regulation by the RNA binding protein LIN28 [13], and the RNA editing enzyme ADAR [14] (Figure 1).

An important let-7 target is the RAS family, regulating K-RAS mRNA and therefore the cell proliferation is reduced upon let-7d upregulation. Other targets are MYC, IMP-1 and High Mobility Group A2 (HMGA2) [15]. In line with this tumor-suppressive role, let-7 family members are de-regulated in several human cancers. The expression of let-7d is low in cancer as pancreatic, prostate, primary pigmented nodular adrenal dysplasia, head and neck, bladder, and kidney cancer [16,17,18]. In a therapeutic context, after irradiation, let-7 family members can be up- and downregulated, which depends on dosage, time after the irradiation, source of oxidative stress and genetic background of the cell [19,20]. Apart from acting on tumor cells, let-7d may also regulate the inflammatory tumor microenvironment, as an immunoregulatory role has been demonstrated for this microRNA. For example, transcriptional analysis and microRNA inhibitor studies have demonstrated that exosomal delivery of Let-7d from Treg cells to Th1 cells was able to suppress systemic inflammatory disease in a mouse model [21]. Moreover, studies in knockout mice revealed that the let-7 cluster (including let-7d) suppresses B cell activation via a metabolic mechanism that resulted in restriction of necessary nutrients, thereby affecting B-cell dependent antibody production [22]. Finally, let-7d targets the epigenetic regulator Tet2, which demethylates deoxycytosine residues in DNA, resulting in enhanced expression of IL-1ß and IL-6 in macrophages in murine models of inflammation [23]. Overall, these studies emphasize the relevance of let-7d as an important suppressor of tumor progression and regulator of inflammatory processes. In the following sections, we will highlight the role of dysregulated let-7d expression in female malignancies and diseases of the female reproductive tract (Figure 1).

## 3. Let-7d and Breast Cancer

Breast cancer is the most frequent cancer among all women and it is recognized as the second leading cause of cancer deaths [10,25]. Dysregulated expression of microRNAs in breast cancer has been mechanistically linked to Hallmark processes of cancer [3,26,27,28], whereas altered expression of selected microRNAs is associated with prognosis and therapeutic response [29,30,31,32].

In the past, gene expression studies demonstrated that estrogen receptor (ER)—positive and ER-negative breast cancer are different diseases and that progesterone receptor (PR) and HER2 expression were important for classification and therapy. ER-/HER2- negative breast cancer is an aggressive cancer not responsive to targeted treatment [33]. Selected microRNAs are associated with distinct subtypes of breast cancer. For ER-/HER2- breast cancer, Lee et al. postulated the activities of microRNA let-7d and miR-18a as possible prognostic factors and confirmed this assumption in two independent ER-/HER2- breast cancer gene expression databases: the overactive let-7d was associated with a better overall survival rate, whereas in one dataset, the activities of let-7d were related to the metastatic process [34]. Moreover, the genetic variant rs13293512 in the promoter of the let-7a1/f1/d cluster was found to be associated with an increased risk of breast cancer in Chinese women, in particular in the estrogen receptor-negative, the progesterone receptor-negative, and nodal positive patient subgroups [35]. Indeed, let-7 family members are expressed in an estrogen-dependent manner in breast cancer, providing a possible explanation for hormone-receptor-associated differences in the prognostic and diagnostic value of this microRNA family. For example, a study in estrogen-regulated miRNAs in this tumor entity revealed that treatment of MCF-7 breast cancer cells with estradiol (E2) induced the expression of let-7d and seven additional let-7 family members [36]. Among those, several E2-regulated microRNA genes were either associated with ERa-binding sites or located in intragenic regions of estrogen-regulated genes. In turn, let-7 family members target ERalpha via interactions with the 3′UTR of ERalpha mRNA, and do thereby enhance the sensitivity of MCF-7 breast cancer cells to tamoxifen therapy [37,38,39]. Further studies indicated that this modulation of estrogen sensitivity by let-7c affected the Wnt-signaling pathway, with implications for inhibiting the cancer stem cell population with tamoxifen [40,41]. Notably, it was shown that another let-7 member, let-7a, was expressed depending on the androgen receptor, providing an additional example for steroidal regulation of let-7 in breast cancer [42]. It remains to be shown to which extent the observed effects are isoform-specific or also of potential relevance for let-7d.

A deep sequencing study of patient tissues revealed that microRNA regulates the transition process from the normal breast to ductal carcinoma in situ and then to invasive ductal carcinoma. Let-7d, along with miR-210 and miR-221 was part of a microRNA signature associated with this process, as it is downregulated in in situ carcinoma and up-regulated in invasive transition [43]. LIN28 is an RNA-binding protein, its role is the inhibition of premature let-7 precursor processing. It controls the maturation of all let-7 family members and thereby regulates cellular differentiation. Based on the inverse correlation of let-7d and other let-7 members with the RNA binding protein LIN28 in breast cancer, it has been suggested that LIN28 promotes tumorigenic activity by suppressing let-7 microRNA maturation in breast carcinoma cells [44]. At the mechanistic level, LIN28 protein binds to the terminal loop of primary and precursor forms of let-7 and thereby represses their processing by Drosha and Dicer, which in turn affects the posttranscriptional regulation of let-7 targets [13,44,45]. In turn, let-7 is able to repress the post-transcriptional translation of LIN28, thus suggesting the presence of a double-feedback loop of LIN28/let-7 regulation [46,47]. This regulation is of major importance in an oncological context, as LIN28 promotes transformation in vitro and is associated with advanced cancer in several tumor entities, including breast and cervical cancer [48]. A de-repression of oncogenic let-7 targets, including K-Ras, c-Myc, and the DNA binding protein HMGA2, is part of the tumor-promoting activity of LIN28 [45,49].

In the past, progress was made in diagnostics of breast cancer in early stages, but the development of metastases remains the main predictor of mortality in patients with breast cancer [10,50]. Several processes are involved in the development of metastasis, including local invasion, tumor cell migration, dissemination via the bloodstream and lymphatics and colonization at distant sites. One of the central elements for metastatic diffusion is the process of epithelial–mesenchymal transition (EMT) [3,28,51]. EMT defines a phenomenon describing how cells lose their epithelial characteristics and acquire the motility property of mesenchymal cells [51,52]. Previous 3′UTR luciferase reporter assays using 293T cells had indicated that the small GTPase Rab25 is a direct regulatory target of let-7d [53]. Rab25 plays an important role in regulating vesicular trafficking to the cell surface. Although context-dependent effects have been described, Rab25 can promote tumor progression and aggressive cell behavior by regulating integrin recycling and intracellular signaling pathways, thereby enhancing cell motility and metastasis [54,55]. A recent study extended the relevance of this finding to breast cancer: The authors demonstrated that the expression of let-7d and Rab25 were inversely correlated in a study on 110 breast cancer samples and adjacent tissues [56]. Notably, let-7d expression was associated with tumor size, tumor stage and lymph node metastasis. Both Rab25 and the mesenchymal marker snail were upregulated in breast cancer tissue, and were correlated, leading the authors to suggest that let-7d regulated EMT in breast cancer by targeting Rab25 [56].

A study combining expression analysis in patient biopsies with functional in vitro and in vivo investigations demonstrated that let-7d regulates cell growth and invasion in breast cancer, and also inhibits Jab1 (Jun activation domain-binding protein 1) protein expression [57]. Jab1 is a component of the COP9 signalosome protein complex that regulates fundamental cellular processes linked to the ubiquitin–proteasome system [58]. It acts as a docking interface for protein kinases, thereby affecting numerous signaling pathways relevant to tumor progression, including p53, p27 AP-1 and Smad proteins active in TGF-β1 signaling [59,60,61]. A worse prognosis for breast cancer was significantly correlated with low levels of let-7d or high levels of Jab1 [57]. In this study, patients with high let-7d cancers had a longer mean survival time (113 months) than the patients with low tumoral let-7d expression. Dual-luciferase reporter assays confirmed that Jab1 was a direct regulatory target of let-7d, whereas in vitro assays on MDA-MB-231 and MCF-7 cells and xenograft in vivo assays demonstrated a role for let-7d in regulating breast cancer cell proliferation, invasion and tumor growth in vivo [57]. Let-7d was also shown to play a role in the development of breast cancer metastasis to the brain [62]. The loss of let-7d and activation of hypoxia-inducible factor-1 signaling induced brain metastasis via a platelet-derived growth factor (PDGF) pathway in a mouse model of spontaneous breast cancer metastasis from the primary site to the brain: If PDGFR was pharmacologically inhibited, experimental brain metastasis was suppressed, pointing out new therapeutic opportunities [62]. An example of another therapeutic approach is the analysis of microRNA as possible indicators of drug sensitivity. In an in vitro study associating IC50 values of 34 with microRNA expression data of human breast cancer cell lines, let-7d expression was associated with a high sensitivity to Tivantinib [63]. Tivantinib is an orally available, small-molecule, non-AT competitive c-MET inhibitor that is highly specific for the c-MET receptor. The disruption of hepatocyte growth factor (HGF)/c-MET signaling is a potential targeted approach to treating metastatic bone disease, and the combinatorial use of Tivantinib and zoledronic acid has yielded promising therapeutic results in a preclinical animal model of bone metastasis of breast cancer [64]. However, overall, only limited data exist regarding its evaluation in further gynaecological malignancies. A phase II clinical trial in metastatic triple-negative breast cancer did not meet pre-specified statistical targets for efficacy [65]. It remains to be shown if a subgroup of patients displaying high tumoral let-7d expression may benefit to a larger extent from Tivantinib treatment.

Apart from pharmacotherapy, let-7d was also shown to sensitize breast cancer stem cells to radiotherapy by inhibiting the cyclin D1/Akt1/Wnt1 signaling pathway, extending the relevance of this microRNA to further modes of therapeutic intervention [66]. Overall, there results suggest that pharmacological modulation of let-7d levels may represent a worthwhile therapeutic concept in future breast cancer therapies.

## 4. Let-7d and Ovarian Cancer

Ovarian cancer is the seventh most frequent cancer and the eighth main cause of cancer-associated mortality [67]. The prognosis is poor: In the majority of cases the disease is in an advanced state at the moment of the diagnosis and the overall 5-year survival rate is only about 50%. Ovarian cancer has different histologic types; the most frequent is epithelial ovarian cancer (with the subtypes: serous, endometroid, mucinous and clear cell). Most of the patients have high-grade serous ovarian cancer, distinguished by an aggressive development and poor prognosis [68]. Surgery is important not only for the diagnosis and staging of ovarian cancer, but also in the treatment management of patients with advanced disease [69]. Platinum-based chemotherapy (including cisplatin and carboplatin) is the first-line agent in the treatment of ovarian cancer. Metastases are the main reason for the high mortality in these patients [70]. Studies in model organisms have revealed a gradual increase in ovarian and pituitary let-7 expression, including let-7d, during ovarian development, suggesting a potential role of this microRNA in ovarian function [71].

Similar to other tumor entities, aberrant expression of let-7 family members in patients with ovarian cancer compared with that in healthy controls was identified in several studies and so the differentially expressed microRNA may have potential as a diagnostic marker [72,73,74]. However, let-7 dysregulation showed a heterogenous pattern, depending on the subtype and family member involved. For example, in liquid biopsies, let-7b is upregulated, whereas let-7f and let-7i are downregulated in ovarian cancer [75]. Among all let-7 family members, also let-7d was found to be dysregulated in ovarian cancer [76], and it has been shown that the cytokine PDGF-AA is capable of repressing let-7d expression in ovarian cancer cells [77]. High throughput sequencing revealed that the level of plasma exosomal let-7d-5p in patients with ovarian cancer was significantly higher compared to the controls [72]. Similarly, let-7d-3p was upregulated in ovarian cancer tissue relative to normal ovarian tissues in a study assessing its role in the response to neoadjuvant chemotherapy [73]. Moreover, serum levels of let-7d-3p were found to be able to discriminate epithelial ovarian cancer patients from healthy controls [74]. In the same study, HMGA2 and Kirsten Rat Sarcoma Viral Oncogene Homolog (KRAS) were identified as predicted targets of let-7d by bioinformatics analysis, and their expression levels also had a diagnostic value in the studied patient collective [74]. HMGA2 regulates gene expression by binding to AT-rich regions of DNA and thereby promotes tumor progression via different mechanisms. These include the promotion of a cancer stem cell phenotype, improved DNA repair mechanisms linked to therapeutic resistance, and regulation of multiple signaling processes that drive prometastatic EMT [78,79,80]. KRAS also has an ongogenic function in ovarian cancer, as constitutive activation of this small GTPase by gene mutations activates uncontrolled ovarian cancer cell proliferation via activation of the mitogen axctivated protein kinase (MAPK)/ extracellular signal regulated kinase (ERK) pathway, a signaling pathway that is also regulated by HMGA2 [78,81]. Therefore, downregulation of HMGA2 and KRAS by let-7d could exert an anti-oncogenic effect in ovarian cancer. However, a study comparing epithelial ovarian cancer cell lines and immortalized ovarian surface epithelium cell lines reported a significant decrease of let-7d in the cancer cells, suggesting potential cell-type- or context-dependent variability regarding let-7d expression in this tumor entity [82]. Aberrant let-7d levels are not only seen in ovarian cells, but also in cells of the tumor microenvironment, as let-7d was significantly downregulated in highly inflamed lymphatic vessels infiltrating ovarian tumors compared to less inflamed vessels [83]. Notably, this study revealed that high inflammation correlated with the relapse of ovarian cancer.

A variety of mechanistic studies supports the concept of a specific functional involvement of let-7d in ovarian cancer pathogenesis. Analysis of a panel of patient-derived high-grade serous ovarian cancer cells revealed an inverse association between microRNA let-7 expression and the cancer stem cell phenotype, with low let-7 levels being associated with an epithelial phenotype [84]. This finding may be associated with a regulatory impact of the EMT-marker snail on let-7 [85]. One target of let-7d is c-Myc, an essential oncogenic transcription factor that is involved in tumor pathogenesis and frequently dysregulated in cancer [86]. c-Myc expression is upregulated in ovarian cancer, driving tumor progression by regulating the expression of genes that control tumor cell proliferation, cell-cycle progression, angiogenesis, apoptosis, as well as cell adhesion and motility as a prerequisite for metastatic spread [87]. Let-7d suppresses c-Myc and increases ovarian cancer cell sensitivity to 7-difluoromethoxyl-5,4′-di-n-octylgenistein (DFOG) by inhibiting the PI3K/AKT pathway [88]. DFOG is a synthetic genistein analogue that is under preclinical evaluation. It exerts an anti-ovarian cancer effect by repressing tumor stemness via suppression of PI3K/AKT signaling in vitro and in vivo [88]. In another study, it was demonstrated that an additional target of let-7d is high mobility group A1 (HMGA1) [89]. HMGA1 controls the p53 signaling pathway that is important in the regulation of cell apoptosis and drug resistance at the transcriptional level [90,91]. A putative regulatory axis of let-7d, HMGA2 and KRAS may be associated with tumorigenesis, invasion, and metastasis in epithelial ovarian cancer [74].

Several studies point to a link between let-7d expression and drug response in ovarian cancer [73,89,91]. Indeed, serum levels of let-7a have been proposed as a biomarker for treatment decisions regarding the type of chemotherapy (platinum vs. platinum plus paclitaxel) [75,92]. Considering let-7d, let-7d-5p inhibits ovarian cancer cell motility, cell cycle progression and promotes cell death and cisplatin chemosensitivity by repressing HMGA1 via the p53 pathway [90]. Moreover, an increased expression of let-7d-3p was associated with a better response to carboplatin/paclitaxel treatment in ovarian cancer patients [73]. Functional evaluation of let-7d-3p in SKOV3 ovarian cancer cells in this study revealed that its inhibition impaired cell proliferation and activated apoptosis, but did not affect cell motility and invasiveness. While these studies point at an important role for let-7d dysregulation in ovarian cancer pathogenesis, some context-dependent effects of let-7d function need to be taken into account to evaluate the side effects of potential let-7d-centered therapeutic approaches.

## 5. Let-7d in Additional Gynaecological Malignancies

**Figure 2:**
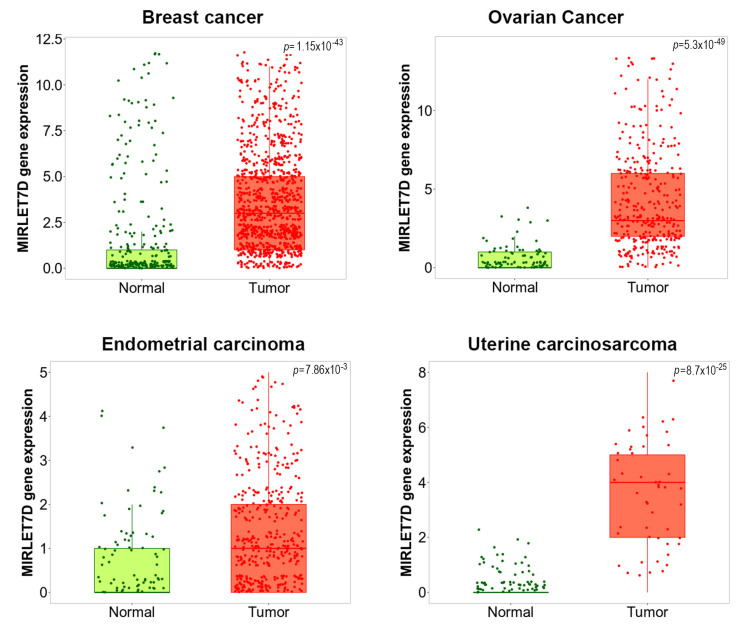
Let-7d is upregulated in a variety of gynecological malignancies. RNASeq-based let-7d expression data were retrieved from the TNMPlot database [93]. In the box plots, bars show the proportions of tumor samples displaying higher expression of the selected gene compared to normal samples at each of the quantile cutoff values (minimum, 1st quartile, median, 3rd quartile, maximum). *p*-values below 0.05 indicate significant differences as assessed by Mann–Whitney test. Significant let-7d upregulation between normal and tumor tissues was observed for invasive breast carcinoma (“Breast cancer”, 403 normal/1097 tumor samples), ovarian serous cystadenocarcinoma (“Ovarian cancer”, 133 normal/374 tumor samples), uterine corpus endometrial carcinoma (“Endometrial cancer” 146 normal/547 tumor samples), and uterine carcinosarcoma (111 normal/56 tumor samples). See reference [93] for a description of retrieved gene expression data and patient collectives.

MicroRNA let-7d also takes part in other gynaecological diseases. We utilized the TNMPLot database [93] to assess let-7d expression in several gynaecological malignancies. As shown in Figure 2, let-7d was significantly upregulated in breast cancer, ovarian cancer, uterine corpus endometrial carcinoma and uterine carcinosarcoma compared to healthy control tissue, indicating its clinicopathological relevance. Indeed, endometrial cancer is the sixth most common cancer in women [94]. Similar to the situation in breast cancer, a study demonstrated that E2 was able to induce let-7-members, including let-7d, in endometrial carcinoma cells. At the functional level, this upregulation was associated with a shift of the apoptosis-related BCL2/BAX protein ratio towards improved cancer cell survival, which would be in line with our in silico data [95]. The authors discussed E2-dependent regulation of let-7 as a possible indicator of estrogen over-exposure.

Another gynecological malignancy of high clinical relevance is cervical cancer, the fourth most common cancer type in women [96]. Plasma exosomal microRNA sequencing identified let-7d as part of an 8 microRNA signature that could discriminate CIN II+ from CIN I- cervical carcinoma patients [97]. Let-7d may serve as a diagnostic biomarker for non-invasive screening of cervical cancer, because let-7d-3p levels differed significantly in healthy tissue and tissue of cervical cancer tissue and adjacent healthy tissue in a validation dataset [97]. Notably, the expression of let-7d in cervical carcinoma cells depends on the expression of human papillomavirus (HPV) E6/E7 oncogene, suggesting a possible partial contribution of this miRNA to HPV-induced cervical carcinoma [98]. Moreover, a study on 34 leiomyosarcomas and 13 normal myometrium paraffin-embedded samples revealed that low levels of let-7d in leiomyosarcoma were associated with shorter disease-free survival [99].

## 6. Let-7d in Endometriosis

Endometriosis is a disease characterized by ectopic growth of endometrial tissue, resulting in pain symptoms and reduced fertility in affected patients [100]. MicroRNA expression is dysregulated in endometriosis and has been functionally linked to invasive growth, aberrant proliferation and stem cell function [101,102,103,104]. MicroRNA let-7d-3p is significantly downregulated in women with endometriosis [105]. When combined with microRNAs miR-199b-3p and miR-224-5p, circulating microRNA let-7d-3p levels can discriminate endometriosis patients from non-endometriosis women with a sensitivity of 96% and specificity of 100%, respectively. An independent study revealed that a combination of the serum levels of let-7b,-7d and 7f during the proliferative phase could be a diagnostic marker of endometriosis [106]. 

## 7. Let-7d in Pregnancy Complications

Altered expression of let-7d is associated with pregnancy complications. The importance of this microRNA for uterine function was highlighted in studies on mice exhibiting a tissue-specific gene knockout of the microRNA processing enzyme Dicer: Using a progesterone receptor-Cre construct, Dicer was conditionally knocked out in postnatal uterine epithelium and stroma, resulting in sterile female mice with small uteri [107]. At the functional level, the absence of glandular epithelium and enhanced stromal apoptosis in the uteri was associated with a reduction in the levels of let-7d and additional microRNAs (miR-181c, miR-200b, miR-101) and upregulation of pro-apoptotic genes targeted by these microRNAs (Bcl2l11, Aldh1a3), in accordance with the apoptosis phenotype. Furthermore, in vitro studies on the invasion ability and angiogenesis-promoting activity of primary extravillous trophoblasts and the HTR8/SVneo (HTR8) cell line have documented a role for DICER in this process. Notably, the antiangiogenic effect of shRNA-mediated DICER depletion in a HUVEC angiogenesis assay was partially attributed to an increase in antiangiogenic let-7d, highlighting the importance of this microRNA for a successful pregnancy [108]. In line with these findings, let-7d was identified as part of a 27 microRNA signature that was positively associated with pre-pregnancy body mass index as a risk factor for several pregnancy complications and adverse offspring outcomes [109]. Notably, an analysis of the microRNAome of normal pregnant and miscarriage deciduas revealed that let-7d was downregulated along with other let-7 family members in aborted deciduas [110]. Moreover, low circulating plasma levels of let-7d were found to be associated with recurrent pregnancy loss, a major pregnancy complication that affects 2–3% of all pregnancies [111]. Moreover, circulating maternal plasma levels of let-7d-5p were part of a microRNA signature that discriminated between small for gestational age cases and controls at 12-14+6 weeks gestation [112]. let-7d-5p was of diagnostic value both in the discovery and validation cohort of this study, and an AUC of 0.74 was achieved for hsa-let-7d-5p in ROC curve analysis, indicating a value for this microRNA in determining risk for stillbirth or long-term adverse outcomes. A diagnostic value of serum let-7d was also documented in the case of Down syndrome, as let-7d was part of a plasma microRNA signature in women with foetal down syndrome [113].

A severe pregnancy complication that affects both the mother and the fetus is preeclampsia, a multisystem disease linked to high blood pressure [114]. In situ hybridisation analysis of placental tissue from 63 preeclampsia patients and 65 normal control tissues revealed increased expression of let-7d in the diseased tissue. Further functional analysis of trophoblast cells transfected with let-7d inhibitors demonstrated that inhibition of let-7d increased cell proliferation and inhibited apoptosis, along with an upregulation of the invasion-related matrix metalloproteinases MMP-2 and MMP-9 and its inhibitor TIMP-1 [115]. These data suggest that upregulation of let-7d in preeclampsia may have a detrimental effect on trophoblast survival and its capability to invade.

## 8. A Possible Role for Let-7 (d) in Reproductive Aging?

The process of reproductive aging has strong implications for female fertility and reproductive outcome, not only from a clinical, but also from a molecular to a morpho-functional point of view [116]. Indeed, an advanced reproductive-aged female phenotype is associated with altered expression of specific microRNAs, regulating gene expression, chromatin remodelling and early embryo development, as summarized in a recent review [117]. While let-7d or other let-7 family members were not specifically identified in these studies, future studies in this context may be worthwhile, as research on animal models has identified a clear link between let-7 and aging in general. In the worm *C. elegans*, let-7 regulates developmental timing, and low levels of the RNA-binding protein LIN-28 enhance longevity, and reduce germline progenitor cell numbers by regulating let-7, which in turn targets the signaling kinases Akt-1/2 and the downstream transcription factor DAF-16 [118,119]. Notably, in the fruit fly *Drosophila*, let-7 regulates ageing of the testis stem-cell niche, indicating its relevance for male reproduction [120]. Finally, studies in knockout mice deficient in the long noncoding RNA H19 have suggested a possible regulatory impact of let-7 on anti-muellerian hormone (AMH): H19 acts as a “sponge” inhibiting let-7 function, and let-7 was demonstrated to target AMH expression [121]. In the mouse model, absence of H19 resulted in subfertility, accelerated follicular recruitment and decreased ovarian AMH levels. As AMH is an important marker of the ovarian reserve and the age-related decline of the ovarian pool, a more detailed investigation of the role of let-7 family members in female reproduction could be a promising approach [122,123].

## 9. Conclusions

Let-7d expression is dysregulated in a variety of gynecologic diseases. In an oncological context, let-7d is under the transcriptional regulation of oncologically relevant transcriptional regulators, including MYC and p53 (Figure 1). Posttranscriptional regulation by the LIN28 and ADAR expands the regulatory repertoire of let-7d. In turn, let-7d acts as a tumor suppressor by synchronously targeting mRNAs involved in the regulation of tumor cell proliferation, apoptosis (resistance), angiogenesis and inflammation, resulting in improved therapeutic response. However, some of the functions of let-7d appear to be context-dependent and are not always fully in line with the tumor suppressor concept, requiring caution upon the development of let-7-centered therapeutic approaches. Apart from gynaecological malignancies, let-7d is dysregulated in endometriosis, and mechanistically associated with uterine development and function. In a pathophysiological context, it has been linked to several pregnancy complications, including recurrent pregnancy loss and preeclampsia, involving mechanisms similar to its action in oncology. Besides a promising use as a blood-based diagnostic and prognostic marker, the pharmacological restoration of proper let-7d levels emerges as a promising future therapeutic concept for a broad range of disorders affecting the wellbeing of women, which is worthy of further and thorough preclinical evaluation.
